# Stigma toward schizophrenia: do all psychiatrists behave the same? Latent profile analysis of a national sample of psychiatrists in Brazil

**DOI:** 10.1186/1471-244X-13-92

**Published:** 2013-03-21

**Authors:** Alexandre Andrade Loch, Francisco Bevilacqua Guarniero, Fabio Lorea Lawson, Michael Pascal Hengartner, Wulf Rössler, Wagner Farid Gattaz, Yuan-Pang Wang

**Affiliations:** 1 Laboratory of Neuroscience (LIM 27), Department and Institute of Psychiatry, Faculty of Medicine, University of São Paulo, R. Dr. Ovídio Pires de Campos, 785, 3º andar, ala norte, sala 1, São Paulo CEP 05403-000, Brazil; 2 Department of General and Social Psychiatry, Psychiatric University Hospital, University of Zurich, Militärstrasse 8, Zurich, 8004, Switzerland; 3 Collegium Helveticum, a Joint Research Institute between the University of Zurich and the Swiss Federal Institute of Technology, Schmelzbergstrasse 25, Zurich, CH-8092, Switzerland

**Keywords:** Social distance, Stereotype, Prejudice, Psychosis, Mental health professionals

## Abstract

**Background:**

An important issue concerning the worldwide fight against stigma is the evaluation of psychiatrists’ beliefs and attitudes toward schizophrenia and mental illness in general. However, there is as yet no consensus on this matter in the literature, and results vary according to the stigma dimension assessed and to the cultural background of the sample. The aim of this investigation was to search for profiles of stigmatizing beliefs related to schizophrenia in a national sample of psychiatrists in Brazil.

**Methods:**

A sample of 1414 psychiatrists were recruited from among those attending the 2009 Brazilian Congress of Psychiatry. A questionnaire was applied in face-to-face interviews. The questionnaire addressed four stigma dimensions, all in reference to individuals with schizophrenia: stereotypes, restrictions, perceived prejudice and social distance. Stigma item scores were included in latent profile analyses; the resulting profiles were entered into multinomial logistic regression models with sociodemographics, in order to identify significant correlates.

**Results:**

Three profiles were identified. The “no stigma” subjects (n = 337) characterized individuals with schizophrenia in a positive light, disagreed with restrictions, and displayed a low level of social distance. The “unobtrusive stigma” subjects (n = 471) were significantly younger and displayed the lowest level of social distance, although most of them agreed with involuntary admission and demonstrated a high level of perceived prejudice. The “great stigma” subjects (n = 606) negatively stereotyped individuals with schizophrenia, agreed with restrictions and scored the highest on the perceived prejudice and social distance dimensions. In comparison with the first two profiles, this last profile comprised a significantly larger number of individuals who were in frequent contact with a family member suffering from a psychiatric disorder, as well as comprising more individuals who had no such family member.

**Conclusions:**

Our study not only provides additional data related to an under-researched area but also reveals that psychiatrists are a heterogeneous group regarding stigma toward schizophrenia. The presence of different stigma profiles should be evaluated in further studies; this could enable anti-stigma initiatives to be specifically designed to effectively target the stigmatizing group.

## Background

According to Goffman [1], stigma is an attribute of “deeply discrediting”, stigmatized persons being recognized by a differentiating “mark” for which they would be devalued. As such, stigma and its corresponding manifestations (e.g., stereotyping, desire for social distance) have been widely studied in their relationship to mental illness. One important aspect of this topic concerns the attitudes and beliefs of psychiatrists regarding their patients; this aspect has proved to be of utmost importance for research, as well as for the planning of anti-stigma campaigns [2]. Theoretically, psychiatrists should act as role models for such campaigns [3]; however, the recognition of them as opinion leaders is debatable, data in the current literature showing that it is still unclear whether psychiatrists stigmatize their patients or not [4].

Lauber et al. [3] found that psychiatrists were more in favor of community psychiatry than is the general population, although the level of social distance toward an individual with schizophrenia, i.e., unwillingness to engage in social situations with the individual in question, did not differ significantly between the two samples. In a subsequent study, those same authors also observed that negative stereotyping of individuals with mental disorders was common among mental health professionals [5]. Another similar study later replicated the finding that social distance scores did not differ significantly between mental health professionals and the general public [6]. In Italy, 85% of the general population and 76% of mental health professionals were found to hold the opinion that people with schizophrenia are unpredictable [7]; nevertheless, all of them voiced rather innocuous opinions regarding restrictions on the civil rights of individuals with the disorder. Arvaniti et al. [8] reported that the psychiatric staff of the Greek University General Hospital demonstrated attitudes toward mental illness that were more positive than were those demonstrated by other health professionals employed at the hospital. However, in a study conducted in Australia, mental health professionals were found to more often believe that schizophrenia has a deteriorating course and to have negative attitudes toward persons with the disorder more often than did the general public [9]. Thus, the available data regarding stigmatizing attitudes held by mental health professionals toward individuals with mental illness is inconclusive, results varying according to the stigma dimension evaluated and to the cultural background of the sample [4,10-12].

Experience has shown us that stigma is resilient, being difficult to eradicate; anti-stigma initiatives are often less than efficacious, especially regarding stigma toward schizophrenia [13-15]. If we consider the fact that psychiatrists play an important role in such initiatives [2,16,17], together with the fact that discrimination on the part of mental health professionals is commonly reported by patients and families [18-20], it is of utmost importance to shed light on the issue of these professionals’ stigmatizing beliefs. In addition, the necessary cross-cultural data from developing countries are not yet available [21,22].

Our study aimed to analyze in detail the degree of stigma toward schizophrenia on the part of psychiatrists in a developing country by identifying possible profiles of stigmatizing attitudes and beliefs in a sample of psychiatrists in Brazil.

## Methods

### Sampling and procedures

The study was conducted during the 2009 Brazilian Congress of Psychiatry, which was held in the city of São Paulo. Attendees primarily included psychiatrists and psychologists, although social workers, nurses, residents, general practitioners, and other mental health professionals from around the country were also in attendance.

A research enterprise was in charge of the data collection, through the use of a standardized questionnaire. Of a total of seventy lay-interviewers provided by the enterprise, fifty were selected, based on their knowledge of the issue, and were trained by the study’s investigators. Training lasted for two full days and consisted of theoretical lessons and role-playing sessions in order to provide a complete understanding of the instrument and its application. During data collection, interviewers were positioned throughout the congress area and invited attendees to take part in the study. Those who agreed to participate completed the questionnaire in a 15-minute face-to-face interview. No personally identifying information was collected; once a participant had completed the questionnaire, a mark was made on his/her badge to avoid double inclusion.

Approximately 6000 individuals attended the event. Although 2549 were invited to participate, 954 (37.5%) refused: 898 declined before the interview had begun; 38 declined once they learned what the topic of the survey was; and 18 failed to complete the questionnaire. Therefore, the initial sample comprised 1595 individuals: 1416 psychiatrists, 68 general practitioners, 44 psychologists, and 67 other professionals. In our data analysis, only the responses of the psychiatrists were included. Two of the psychiatrists were foreigners and were excluded, resulting in a final sample of 1414 Brazilian psychiatrists. The sample characteristics are depicted in Table 1.

**Table 1:** Characteristics of the study sample

**Variable**	**N = 1414 n(%)**
**Age (years)**	43.3 (12.6)*
**Sex (male)**	787 (55.7)
**Marital status**	
**Single**	472 (33.4)
**Married**	765 (54.1)
**Previously married**	177 (12.5)
**Offspring (yes)**	807 (57.1)
**Level of education (none doctoral degree)**	1082 (76.5)
**Contact with family member with psychiatric disorder**	
**No such family member**	468 (33.1)
**Rarely or several times per month**	409 (28.9)
**Several times per week or daily**	537 (38.0)
**Seeking professional help for a psychiatric disorder**	
**Never sought help**	876 (62.0)
**Sought help, did not receive prescription**	180 (12.7)
**Sought help, received prescription**	358 (25.3)
**Place of work**	
**Public hospital**	571 (40.4)
**Private hospital**	439 (31.0)
**Public outpatient institution**	697 (49.3)
**University hospital**	501 (35.4)
**Private office**	1190 (84.2)
**Mental health insurance company**	415 (29.3)

* only for this variable: Mean (Standard deviation).

This study is in compliance with the Helsinki Declaration and was approved by the Research Ethics Committee of the University of Sao Paulo School of Medicine *Hospital das Clínicas*.

### Instruments and measures

We applied a questionnaire that has been used in similar attitude surveys in Brazil and in other countries [5,6,23-26]. In addition to sociodemographic data (age, sex, marital status, offspring), personal contact with mental illness was assessed: having a family member with psychiatric disorder and the frequency of contact with him/her; having sought treatment for a psychiatric disorder/having been given a prescription for psychotropic drugs. Information on the respondent’s professional practice was also recorded (place of work, time since end of residency in psychiatry, academic background).

The instrument addressed four stigma dimensions, all in reference to individuals with stable schizophrenia. Twelve items assessed stereotyping, three items evaluated restrictions to civil rights, eight items measured perceived prejudice [27], and seven items were adapted from the Social Distance Scale [28].

Regarding stereotypes, participants answered a 3-point Likert scale on how often various characteristics were present in an individual with schizophrenia compared with someone in the general population (1 = “less present”, 2 = “equally present”, 3 = “more present”). There were five positive stereotypes (creative, healthy, self-controlled, gifted, reasonable) and seven negative stereotypes (dangerous, unpredictable, stupid, bedraggled, abnormal, unreliable, weird). Concerning restrictions, participants were asked to answer “yes” or “no” in agreement/disagreement with three ideas: involuntary admission, restriction of voting rights, and revocation of driver’s licenses. The perceived prejudice dimension measured general social attitudes toward persons with schizophrenia. Responses were assessed on a 3-point scale (1 = “I totally disagree”, 2 = “I partly agree”, 3 = “I totally agree”). Items were as follows: “Most people would accept a person with schizophrenia as a close friend”; “Most people believe that someone with schizophrenia is just as intelligent as an average person”; “Most people think that a school teacher with schizophrenia can continue teaching”; “Most people would not allow a person with schizophrenia to take care of their children”; “Most people would hire a person with schizophrenia if her or she was qualified for the job”; “Most people would treat a person with schizophrenia just as they treat anyone else”; “Most people would take the opinion of a person with schizophrenia less seriously”; “Most women would be reluctant to date a man with schizophrenia”. Social distance was measured with an adaptation of the Bogardus Scale [28] and assessed a respondent’s willingness to engage in social interactions with a person with schizophrenia. Participants used a 3-point scale (1 = “certainly”, 2 = “maybe”, 3 = “definitely not”) to demonstrate such willingness in the following situations: moving next door to a person with schizophrenia; allowing a relative to marry a person with schizophrenia; trusting a person with schizophrenia to take care of their children; accepting a job in which they would be working with a person with schizophrenia; introducing a friend to a person with schizophrenia; recommending a person with schizophrenia for a job; and inviting a person with schizophrenia to a party, meeting or dinner.

### Statistical analysis

To identify possible stigma profiles, we opted to use latent profile analysis (LPA) [29], which is similar to latent class analysis; whereas the former can deal with continuous and categorical variables, the latter only handles dichotomous variables. LPA is also based on the principle of conditional independence, which dictates that classes be created such that indicator variables are statistically uncorrelated [30].

Therefore, all 30 items of the four stigma scales were selected as indicators and evaluated for local dependence. Items within a scale were combined in pairs; for each possible pair, we calculated Pearson’s chi-square and residual z-scores for bivariate model fit information (for all possible combinations of that pair’s responses). When more than 50% of the z-scores were above 1.96 or below -1.96, and when Pearson chi-square was above 50,000, local dependence was considered. Clinical judgment decided whether these paired variables should be merged.

The stereotypes “bedraggled”, “abnormal” and “weird” were merged into “strange”, whereas the stereotypes “creative”, “intelligent” and “gifted” were merged into “talented”. For perceived prejudice, “people would think less of” and “people would not hire to take care of their kids” were merged into “think less/not hire to take care of kids”; “people would take his/her opinion less seriously” and “women would be reluctant to date” were also merged into “devaluation/reluctance to date”. For social distance, the variables “recommend for a job”, “allow marriage” and “take care of your children” were merged into “job/marriage/take care of children”. For restrictions, the items were locally independent.

Consequently, the 30 original items were reduced to 23 locally independent items. These were entered into latent profile models to test the most appropriate number of profiles; lowest Bayesian Information Criteria value, highest entropy and a significant Vuong-Lo-Mendell-Rubin likelihood ratio test value served as parameters to indicate the best solution. Conditional probability of membership of each item was computed for the best solution.

Multinomial logistic regression was then conducted to assess factors associated with each profile. In addition to sociodemographic variables, items possibly related to stigma according to the current literature (e.g. personal contact with mental illness, working environment) constituted independent variables. As such, the following 13 variables were entered in the model: age (≤ 30 years; 31–40 years; 41–50 years; 51–60 years; ≥ 61 years), sex (male vs. female), marital status (single; married; previously married), having offspring (yes vs. no), education (no doctoral degree vs. doctoral or post-doctoral degree), frequency of contact with a family member with psychiatric disorder (no such family member; rarely or several times per month; several times per week or daily), having sought professional help for a psychiatric disorder/having been given a prescription for psychotropic drugs (never sought help; sought help, did not receive prescription; sought help, received prescription), place of work (public hospital, private hospital, public outpatient institution, university hospital, private office, mental health insurance—yes vs. no). Analyses were conducted with PASW Statistics 18 and Mplus 5 for Windows. All tests were two-tailed, with a level of significance of .05.

## Results

The three classes clustering was the best solution, which presented the lowest Bayesian Information Criteria values, showed the best entropy and was the highest number of classes with a significant likelihood ratio (Table 2). Tables 3 and 4 indicate the conditional probability of each response-item in the respective profile.

**Table 2:** Latent profile analysis of psychiatrists’ responses to questions regarding stigma

	**BIC**	**Entropy**	**VLMR likelihood ratio**
			***P***
**2 classes**	44481.858	0.660	<.001
**3 classes**	44165.301	0.703	<.001
**4 classes**	44210.297	0.694	.0705

Abbreviations: *BIC *Bayesian Information Criteria; *VLMR* Vuong-Lo-Mendell-Rubin.

**Table 3:** Class size and characteristics, according to conditional probability of individuals in each response item (Stereotypes and Restrictions)

**Stigma dimension**			**Profile 1**	**Profile 2**	**Profile 3**
			**“no stigma”**	**“unobtrusive stigma”**	**“great stigma”**
			**(n = 337)**	**(n = 471)**	**(n = 606)**
	**Item**	**Response**^**a**^	**(%)**	**(%)**	**(%)**
**Negative stereotypes**	Dangerous	More	12.8	8.5	**35.9**
		Equally	69.0	**80.2**	54.1
		Less	**18.2**	11.3	10.0
	Unpredictable	More	47.5	38.7	**84.8**
		Equally	38.5	**56.0**	8.6
		Less	**14.0**	5.3	6.6
	Strange	More	36.2	38.9	**72.9**
		Equally	48.1	**55.8**	16.9
		Less	**15.7**	5.3	10.2
	Untrustworthy	More	6.5	2.2	**29.2**
		Equally	78.9	**92.3**	57.0
		Less	**14.6**	5.5	13.8
**Positive Stereotypes**	Healthy	Less	44.9	56.9	**81.2**
		Equally	**47.6**	39.4	15.2
		More	**7.5**	3.7	3.6
	Controlled	Less	44.9	40.4	**82.9**
		Equally	46.5	**55.3**	11.1
		More	**8.6**	4.3	6.0
	Talented	Less	8.0	7.0	**24.8**
		Equally	82.2	**88.1**	64.2
		More	9.8	4.9	**11.0**
	Rational	Less	33.9	35.0	**80.0**
		Equally	57.7	**58.8**	13.2
		More	**8.4**	6.2	6.8
**Restrictions**	Involuntary admission	Agrees	94.5	**99.4**	97.5
		Disagrees	**5.5**	0.6	2.5
	Should not have a driver’s license	Agrees	2.7	0.9	**12.4**
		Disagrees	97.3	**99.1**	87.6
	Should not have the right to vote	Agrees	13.4	13.7	**41.2**
		Disagrees	**86.6**	86.3	58.8

**Bold**: highest percentage on the line.

^**a**^The first response-item always corresponds to the most stigmatizing answer.

Note: between-group differences were statistical significant for all items (*P* < .001).

**Table 4:** Class size and characteristics, according to conditional probability of individuals in each response item (Perceived prejudice and Social Distance)

**Stigma dimension**			**Profile 1**	**Profile 2**	**Profile 3**
			**“no stigma”**	**“unobtrusive stigma”**	**“great stigma”**
			**(n = 337)**	**(n = 471)**	**(n = 606)**
	**Item**	**Response**^**a**^	**(%)**	**(%)**	**(%)**
**Perceived prejudice**	Accept as a close friend	Disagree	22.7	**87.0**	73.0
		Partially agree	**67.2**	10.9	22.5
		Agree	**10.1**	2.1	4.5
	Just as intelligent as the average person	Disagree	27.4	**79.0**	60.9
		Partially agree	**48.2**	17.8	32.1
		Agree	**24.4**	3.2	7.0
	Accept as a teacher of young children	Disagree	40.0	**92.8**	78.5
		Partially agree	**46.6**	4.5	16.2
		Agree	**13.4**	2.7	5.3
	Think less of/not hire to take care of kids	Agree	55.6	**84.3**	83.0
		Partially agree	**40.5**	14.6	15.5
		Disagree	**3.9**	1.1	1.5
	Hire for a job	Disagree	29.8	**79.6**	67.0
		Partially agree	**49.3**	18.5	28.5
		Agree	**20.9**	1.9	4.5
	Treat him/her equally	Disagree	46.5	**92.1**	85.8
		Partially agree	**46.1**	6.2	12.5
		Agree	**7.4**	1.7	1.7
	Devaluation/reluctance to date	Agree	55.3	**82.0**	80.7
		Partially agree	**43.2**	16.6	18.5
		Disagree	**1.5**	1.4	0.8
**Social distance**	Work	Never	0.0	0.2	**2.1**
		Maybe	13.1	10.4	**29.7**
		Yes	86.9	**89.4**	68.2
	Live as neighbor	Never	0.6	1.3	**7.9**
		Maybe	17.0	7.4	**31.8**
		Yes	82.4	**91.3**	60.3
	Introduce a friend	Never	0.0	0.0	**3.6**
		Maybe	8.6	5.7	**15.3**
		Yes	91.4	**94.3**	81.1
	Meet (dinner, party, etc.)	Never	0.3	0.4	**1.5**
		Maybe	14.8	7.7	**31.4**
		Yes	84.9	**91.9**	67.1
	Job/marriage/take care of children	Never	4.2	3.6	**21.1**
		Maybe	59.1	54.8	**70.5**
		Yes	36.7	**41.6**	8.4

**Bold**: highest percentage on the line.

^**a**^The first response-item always corresponds to the most stigmatizing answer.

Note: between-group differences were statistical significant for all items (*P* < .001).

Profile 1 comprised 337 individuals (23.8% of the sample), with a mean age of 45.8 years. Comparing these individuals with psychiatrists of the other profiles, the profile 1 subjects were the ones that most ascertained that negative stereotypes are less present in the individual with schizophrenia than in the general population (14.0-18.2% vs. 5.3-13.8%). For positive stereotypes, they were the ones who most often selected the “more present” option (7.5%-8.6% vs. 3.6-6.8), the exception being “talented” (9.8% vs. 4.9-11.0%). Regarding restrictions, they had the highest proportional disagreement with involuntary admission (5.5% vs. 0.6-2.5%) and with restricting voting rights (86.6% vs. 58.8-86.3%). Considering perceived prejudice, they mostly responded “partially agree” to the statements (40.5-67.2% vs. 4.5-32.1%) and were the ones who most tended to believe society does not stigmatize individuals with schizophrenia (‘society does not stigmatize’ scores = 1.5-24.4% vs. 0.8-7.0%). Social distance showed intermediate scores in comparison with the other profiles, but was overall low, with “yes” responses predominating (82.4-91.4%), the exception being “job/marriage/take care of children” (“maybe” = 59.1%). For the positive beliefs of the individuals it comprised, this profile was designated “no stigma”.

Profile 2 comprised 471 individuals (33.3% of the sample), with a mean age of 40.8 years. Comparing this profile with the others, negative stereotypes (55.8-92.3% vs. 8.6-78.9%) and positive stereotypes (55.3-88.1% vs. 11.1-82.2%) both received the highest rates of the “equally present” option, the exception being “healthy” (“equally present” = 47.6% for profile 1 vs. 39.4% for profile 2). Regarding restrictions, the profile 2 subjects were the ones who most often agreed with involuntary admission (99.4% vs. 94.5-97.5%). For perceived prejudice, they were the ones who most often believed that the population had stigmatizing views on schizophrenia (79.0-92.8% vs. 22.7-85.8%). Concerning social distance, they demonstrated the highest willingness to engage in social situations with individuals with schizophrenia in comparison with the other profiles (41.6-94.3% vs. 8.4-91.4%). Due to the neutral stereotypes characterization and to the discrepant findings in other dimensions, this profile was designated “unobtrusive stigma”.

Profile 3 comprised 606 individuals, making it proportionally the largest group (42.3% of the sample), with a mean age of 43.9 years. Compared with those of the other profiles, the profile 3 subjects were the ones who most often endorsed the idea that negative stereotypes were more present (29.2-84.8% vs. 2.2-47.5%) and positive stereotypes were less present (24.8-82.9% vs. 7.0-56.9%) in individuals with schizophrenia. They were the ones that most agreed with revoking driver’s licenses (12.4% vs. 0.9-2.7%) and restricting voting rights (41.2% vs. 13.4-13.7%). In terms of social distance, they were also the ones who most often selected the option of “never” engaging in the specified social situations (1.5-21.1% vs. 0.0-4.2%) and who least often responded with a “yes” (8.4-81.1% vs. 36.7-94.3%). Compared with those of the other profiles, the profile 3 psychiatrists showed intermediate scores for perceived prejudice, although, in general, they believed that society stigmatizes individuals with schizophrenia (60.9-85.8%). Accordingly, this profile was designated “great stigma”.

Pearson chi-square statistics showed that scores on all the above mentioned items were statistically different between profiles (*P* < .001). Mean age was also statistically different between groups, as calculated by analysis of variance (f = 16.952; *P* < .001, data not shown).

Results of the multinomial logistic regression are shown in Table 5. Taking the “no stigma” profile as the reference class, we found that individuals in the “unobtrusive stigma” profile were significantly younger, ages below 50 years showing a statistically significant correlation with inclusion in the profile (OR = 2.61-4.00). Regarding the “great stigma” profile, being 31–40 years of age was significantly correlated with inclusion in the profile (OR = 1.79, 95% CI [1.08, 2.97]) and having “rare contact” with a family member with mental illness was protective of membership in this profile (OR = 0.71, 95% CI [0.50, 0.99]). None of the other factors were significantly correlated with the profile.

**Table 5:** Multinomial logistic regression between sociodemographic variables and profiles, adjusted models (N = 1414)

**Variable**	**Profile 2 – Unobtrusive stigma**		**Profile 3 – Great stigma**	
	**OR (95% CI)**	***P***	**OR (95% CI)**	***P***
Age (years)				
18-30	**4.00 (1.98-8.09)**	**<.001**	1.25 (0.66-2.35)	0.49
31-40	**3.50 (1.93-6.35)**	**<.001**	**1.79 (1.08-2.97)**	**<.05**
41-50	**2.61 (1.48-4.60)**	**<.001**	1.31 (0.81-2.12)	0.26
51-60	1.59 (0.91-2.80)	0.11	0.94 (0.59-1.50)	0.79
≥ 61	ref.		ref.	
Contact with family member with mental disorder				
No such family member	*		0.80 (0.58-1.12)	0.19
Rare contact			**0.71 (0.50-0.99)**	**<.05**
Frequent contact			ref.	

NOTE: profile 1 was the reference profile for the regression models.

**Bold:** significant associations.

*Was not in the final model.

## Discussion

This is the first study to analyze psychiatrists’ stigma toward schizophrenia utilizing the LPA technique. To our knowledge, this is also the first time a large sample of mental health professionals has been assessed regarding this issue in such an under-researched area, a Latin American country. We were able to identify three distinct stigma profiles within the group of Brazilian psychiatrist studied.

The “no stigma” profile was the smallest group. The “no stigma” psychiatrists positively stereotyped individuals with schizophrenia, showed the highest disagreement with restrictions, and had the best impression on society’s stigma toward schizophrenia. The “unobtrusive stigma” psychiatrists were significantly younger; they matched individuals with schizophrenia and the general population regarding stereotypes. Although they displayed the lowest level of social distance, they mostly agreed with involuntary admission, showing also the highest perceived prejudice. The majority of psychiatrists in our sample were categorized as belonging to the “great stigma” profile. They negatively stereotyped individuals with schizophrenia, they mostly agreed with restrictions, and they demonstrated the highest level of social distance. They were significantly overrepresented by individuals between 31 and 40 years of age and were significantly less likely to have had only rare contact with a family member with a psychiatric disorder.

Although most of the data in the literature shows that mental health professionals might stigmatize their patients, there have been some reports to the contrary [4]. We argue that an important factor to be put into perspective regarding this issue is the heterogeneity of the professional class, especially of psychiatrists [31]. Previous studies of the same class have demonstrated that, in general, psychiatrists hold stigmatizing beliefs about individuals with schizophrenia [32]. However, further investigation revealed distinct patterns of the psychiatrists’ stigma; not all of them showed negative beliefs, with nearly one quarter of the sample indicating innocuous opinions. Compared with those of the other profiles, the “no stigma” individuals were significantly older; this might illustrate the hypothesis that contact with individuals with mental disorder, here derived from a longer professional experience, diminishes stigma [33]. Lewis et al. [34] also found that older psychiatrists predicted less violence and a better outcome for someone with psychosis than did their younger colleagues. It might be also possible that this could represent the different settings in which these psychiatrists were trained [35,36]. Younger psychiatrists have had greater access to the biological basis of mental disorders, a type of knowledge that usually increases the risk of nurturing stigma [37-40].

The “unobtrusive stigma” group comprised individuals with uncertain opinions. Based on the low level of social distance and on the neutral stereotypes attribution, one hypothesis would be that the psychiatrists in this profile truly believe that individuals with schizophrenia are like anyone else. High perceived prejudice would thus represent their genuine impression of society’s stigma. Nevertheless, these answers could also have been influenced by the “social desirability” response bias [41]. Given that “perceived prejudice” is a way of indirect questioning [42], its high scores could also be an indicator of psychiatrists’ own prejudiced attitudes [4,28]. Griffiths et al. [43] also observed that perceived stigma was higher than was personal stigma, hypothesizing that social desirability makes respondents score low on questionnaires directly addressing their own opinions, while causing them to project their personal stigmatizing beliefs onto others through high perceived prejudice. Considering this interpretation, neutral answers on stereotypes would represent a “central tendency bias” [44], a behaviour often observed while assessing polemic issues [45,46]. The fact that agreement with involuntary admission was highest for the “unobtrusive stigma” profile raises further suspicion of disguised stigmatizing beliefs on the part of the respondents. Consequently, the overrepresentation of younger individuals in this profile allows us to speculate that either inexperienced psychiatrists truly show less stigma while overestimating society’s stigma or less professional experience increases the willingness to give politically correct answers, the professionals being more concerned with the potential of rejection due to their negative views.

The majority of psychiatrists in our sample were categorized as fitting the “great stigma” profile. They were the ones who showed the worst stigmatizing beliefs in all dimensions, except for perceived prejudice. This is in agreement with previous findings that mental health professionals are often rated as a stigmatizing group by people who seek mental health services [47]. In this profile, there was a significant predominance of individuals 31–40 years of age, a stratum representing psychiatrists that have finished their training an average of 5–10 years prior and are beginning their professional career. We can hypothesise that, compared with older psychiatrists, these subjects lack the contact occasioned by professional experience that could diminish stigma [34]. Conversely, it is possible that they show more stigma than their younger colleagues because they were trained in a decade in which awareness of the stigma issue was not widespread [15]. Data in the literature show that the great majority of stigma studies and anti-stigma campaigns were initiated in the mid-1990s, publications emerging from 2000 onward [48]. One interesting finding was that individuals who were rarely in contact with a family member presenting a psychiatric disorder were significantly underrepresented in this profile. In general, such contact is beneficial against stigma, possibly explaining why people with no such contact show stigma [7]. This might be true as long as contact does not exceed a certain degree; frequent contact was also associated with this high stigma profile. Previous findings report that stigma and desire for social distance increases as situations imply greater social closeness [4,49,50]; it turns out that only those with a certain level of contact, i.e. those with rare contact, were protected against membership in the “great stigma” profile.

This study has certain limitations, including the fact that the sample is not necessarily representative of the population of Brazilian psychiatrists. There could be a selection bias relative to those attending the congress. However, because psychiatrists are usually unavailable to surveys in Brazil [51], interviewing them at the national congress was the best way to gather a large nationwide sample. Nevertheless, as previously mentioned, attrition prior to and during the interview was quite high (37.5%). In addition to underscoring psychiatrists’ low commitment to surveys, this could also represent some resistance to revealing possible personal stigmatizing beliefs. In view of this likely bias, our results should be interpreted with caution. Furthermore, conducting face-to-face interviews could be perceived as less anonymous, thereby leading to a greater response bias toward social desirability. This could hypothetically shift psychiatrist’s scores toward more innocuous opinions and obfuscate true stigmatizing beliefs. Although this was conjectured for the “unobtrusive stigma” profile, the effect might also be present in the other profiles.

## Conclusions

Our study adds important cross-cultural data to the international literature. Schizophrenia is a disorder that typically generates high levels of stigma [52], and there are still few data regarding this issue in under-researched areas such as Brazil, especially among mental health professionals. Acknowledging that Brazilian psychiatrists are a heterogeneous group regarding stigmatizing beliefs has significant consequences, such as for local anti-stigma initiatives. The “unobtrusive stigma” profile merits further investigation, because the impartial scores associated with the profile might be the result of social desirability pressure and resistance to recognizing one’s own negative views [53]. Psychiatrists who are initiating their professional careers need a different and tougher intervention regarding their stigmatizing beliefs, given that most of them fitted the “great stigma” profile. The fact that this profile grouped the majority of psychiatrists also contributes to the view that mental health professionals might constitute an important source of stigma [54]. This bolsters international recommendations that mental health providers should become more aware of their own stigmatizing behaviours [47], and supports the idea that interventions should specifically address them, once they are frequently considered a blind-spot in anti-stigma programmes [4]. Nevertheless, these conclusions should be interpreted with caution in view of the previously mentioned limitations.

Contributing to the worldwide fight against stigma, LPA proved useful in evaluating the large population studied here. Further studies employing LPA are needed in order to achieve the following objectives: to assess whether these three profiles of stigma among psychiatrists are reproducible in different sociocultural backgrounds or are specific to Brazil; to delineate more clearly the groups of mental health professionals that display stigmatizing views and target them more directly, designing specific and more effective anti-stigma initiatives; and to increase our understanding of why results related to this issue vary so much across populations. This might be explained in part by the heterogeneity of psychiatrists’ beliefs, which in our case were clustered into distinct profiles.

## Competing interests

The authors declare that they have no competing interests.

## Authors’ contributions

All of the authors contributed to the conception and design of the study. AAL, FBG, FLL and YPW participated in the acquisition of data. AAL and YPW participated in the data analysis and interpretation. AAL drafted the manuscript. All of the authors were involved in critically revising the manuscript for major intellectual content and approved the final version.

## Pre-publication history

The pre-publication history for this paper can be accessed here:

http://www.biomedcentral.com/1471-244X/13/92/prepub
